# Adsorption of organic contaminants of emerging concern using microalgae-derived hydrochars

**DOI:** 10.1038/s41598-025-92717-y

**Published:** 2025-03-17

**Authors:** Ivan Kozyatnyk, Veronica Benavente, Eva Weidemann, Stina Jansson

**Affiliations:** 1 Department of Chemistry, Umeå University, Linnaeus väg 6, 901 87 Umeå, Sweden; 2 Department of Health, Medicine and Caring Sciences, Unit of Clinical Medicine, Occupational and Environmental Medicine, Linköping University, 581 83 Linköping, Sweden; 3 RISE Processum AB, Hörneborgsvägen 10, 891 22 Örnsköldsvik, Sweden

**Keywords:** Hydrothermal carbonisation, Organic pollutants, Emerging contaminants, Wastewater remediation, Pharmaceuticals, Microalgae, Environmental chemistry, Surface chemistry

## Abstract

This study explored the adsorption capacity of hydrochars derived from a strain of microalgae biomass native to northern Sweden for contaminants of emerging concern (CECs) such as caffeine, chloramphenicol, trimethoprim, carbamazepine, bisphenol A, diclofenac, and triclosan. The findings indicate that the surface functionality of the microalgae-derived hydrochars – a blend of alkane/alkene and aromatic structures, coupled with different oxygen-containing functional groups (hydroxyl, carboxyl, and lactone) – significantly influenced the adsorption of the contaminants. The alkane/alkene and aromatic structures increased with increasing hydrothermal treatment temperature, while the oxygen- and nitrogen-containing groups diminished. Bisphenol A and triclosan, which were the compounds with the highest distribution coefficients, displayed improved adsorption on the hydrochars. The study measured peak adsorption values for the hydrochars processed at 180 °C, which achieved adsorption levels of 25.8 mg g^− 1^ for bisphenol A and 58.8 mg g^− 1^ for triclosan. The hydrochars produced using lower carbonisation temperatures (180 and 220 °C) exhibited enhanced adsorption of positively charged molecules such as trimethoprim, which was attributed to the increased presence of negatively charged oxygen-containing functional groups. Contrastingly, negatively charged molecules such as diclofenac and chloramphenicol demonstrated either low adsorption (2.5 mg g^− 1^ for chloramphenicol on hydrochar prepared at 180 °C) or no adsorption (diclofenac) due to repulsion by the negatively charged functional groups on the surface of the hydrochars.

**Supplementary Information:**

The online version contains supplementary material available at 10.1038/s41598-025-92717-y.

## Introduction

Increasing CO_2_ emissions due to industry and population growth are driving global warming, which is an enormous challenge for both this generation and future ones^1,2^. One promising solution to this issue is returning atmospheric CO_2_ to a non-gaseous state via the cultivation of photosynthetic organisms^3^. Green microalgae have an excellent ability to capture carbon due to its high photosynthesis efficiency and fast growth rate^4,5^. One kg of microalgae biomass is able to recycle equivalent of 1.88 kg CO_2_^6^, and its cultivation can take place on non-arable land or in the ocean. Microalgae cultivation in wastewater with high nutrient content is of interest from both environmental and economic perspectives, as it involves the production of potentially valuable biomass while simultaneously sequestering CO_2_ emissions and performing biotreatment of the wastewater^7,8^. The composition of the wastewater, however, can pose a challenge to the survival of the microalgae, although the hardy algae strains of *Chlorella* and *Scenedesmus*, along with locally isolated strains and mixed cultures, have been successfully implemented in wastewater treatment^7,9^. The growth rate of the microalgae, biomass production efficiency, and wastewater remediation are highly influenced by light conditions and temperature, particularly when open systems (e.g. oval raceway ponds) are used^10^. In subarctic climates with short summer seasons (but long daylight hours) and comparatively low average temperatures, microalgal farming can be challenging. Ferro et al.^11^ showed that three Swedish wild microalgal strains (*Chlorella vulgaris*, *Scenedesmus* sp., and *Desmodesmus* sp.) are able to cope with the challenges posed by the Swedish climate, however^12^.

Microalgae biomass can be valorised through conversion into materials with high carbon contents that can be used as fuel^13^, as well as into catalysts^14^, capacitors^15^, and adsorbents for environmental applications^16,17^. Hydrothermal carbonisation (HTC) is an energy-efficient alternative to pyrolysis^18^, as the latter requires energy-intensive drying^19^, in transforming high-moisture microalgae biomass into carbonaceous materials^20,21^. HTC is a low-temperature (180–300ºC) process in subcritical water under self-generated pressure^22–24^ that results in a hydrochar, a liquid phase, and gases^23,25,26^. Because harvested microalgae typically have a water content of 80–90%, HTC is suitable for low-energy (as compared to drying and pyrolysis) transformation of microalgae into carbon (hydrochars)^27^. Microalgae-derived hydrochars can have porosity and surface functionalities that enable them to be used as adsorbents^28^. Oxygenated functional groups (e.g., carboxylic, lactone, and hydroxylic) can enhance the adsorption capacity of heavy metals through electrostatic attraction, ion exchange, and/or surface complexation^29^.

In recent years, research has focused on contaminants of environmental concern (CECs), and remediation methods in wastewater effluent have been developed based on conventional and advanced treatment processes^30,31^. An effective method of removing CECs from water is adsorption using carbon materials^32,33^. Microalgae-derived hydrochars have been investigated in the context of adsorption of CO_2_^34^ and heavy metals^35,36^, their potential for removing organic CECs from multi-component solutions which better reflects real wastewater conditions remains unexplored. Additionally, the relationship between HTC processing conditions and selective CECs adsorption is not well understood, particularly for hydrochars derived from regional biomass sources. This study addresses these knowledge gaps by investigating how HTC conditions affect hydrochar surface functionality and its impact on simultaneous adsorption of multiple CECs with varying physicochemical properties.

In this study, hydrochars that were produced using a wild strain of microalgae native to northern Sweden that was cultivated in an open pond photobioreactor were assessed for their potential to treat wastewater. The aims of the study were to: (i) evaluate the potential of microalgae-derived hydrochars to remove selected CECs with differing hydrophobicity (caffeine, chloramphenicol, trimethoprim, carbamazepine, bisphenol A, diclofenac, and triclosan) from wastewater; and (ii) investigate how several characteristics (porosity, elemental composition, and surface properties) of the hydrochars affect adsorption.

## Results and discussion

### Morphological characterisation of hydrochar surfaces

**Fig. 1:**
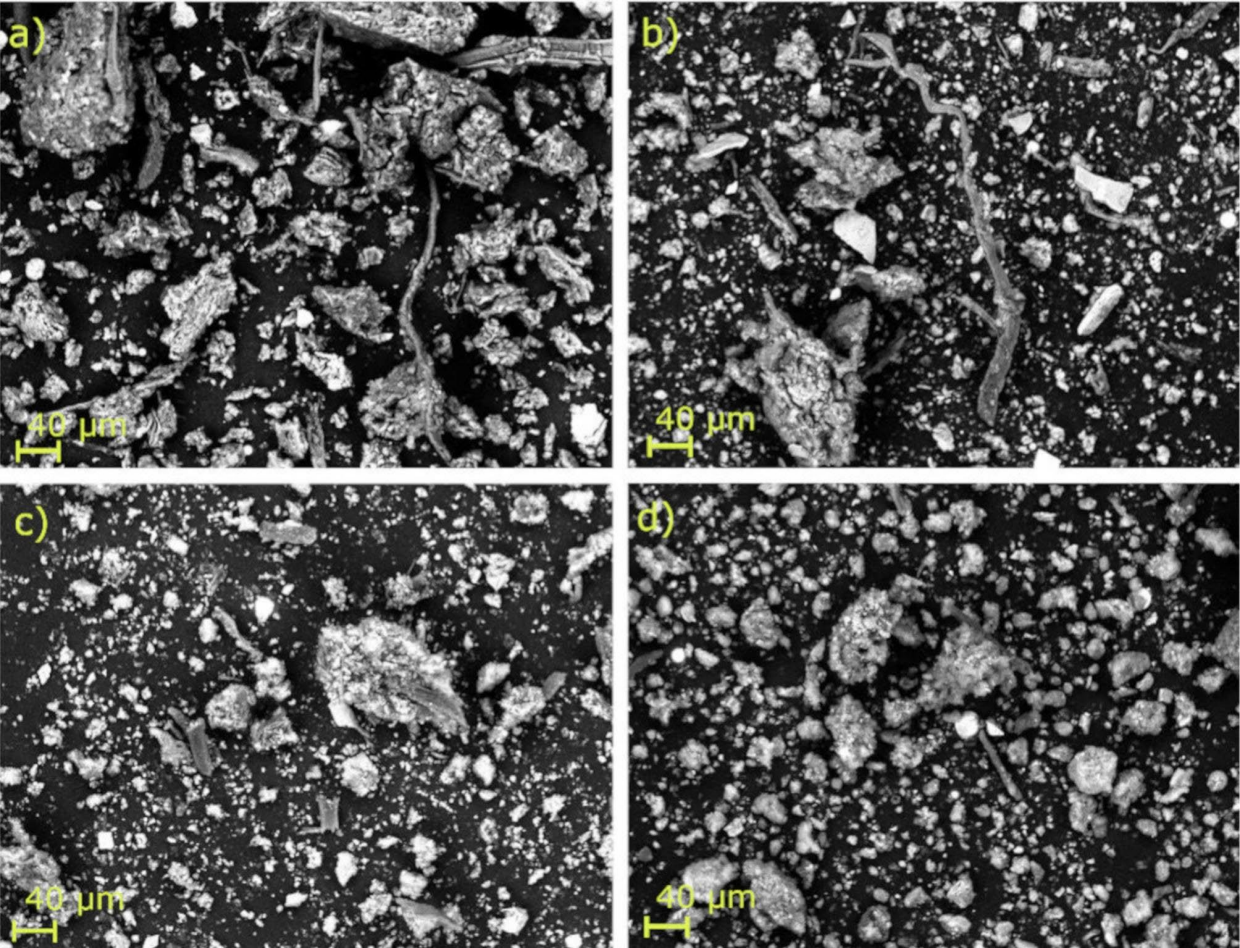
Scanning electron microphotographs of hydrochars prepared from (a) microalgae biomass and hydrochars produced at (b) 180, (c) 220, and (d) 260 °C.

HTC treatment transformed the dark-green microalgae biomass into hydrochars of varying shades of brown. The hydrochar produced at 180 °C had a fibrous structure that was similar to the initial raw algae biomass, while no fibres were visible in that produced at 220 and 260 °C (Fig. 1).

An elevation in HTC temperature covaried with increasing surface area (from 15.3 m^2^ g^− 1^ at 180 °C to 51.2 m^2^ g^− 1^ at 260 °C) and pore volume (from 0.10 cm^3^ g^− 1^ at 180 °C to 0.34 cm^3^ g^− 1^ at 260 °C) (see Table 1 and Fig. S1 in the Supplementary Information). An increase in HTC temperature resulted in a lower hydrochar yield (46% at 180 °C to 26% at 260 °C); the hydrochars produced at higher temperatures were more easily filtered due to the increase in hydrophobicity, as previously described by e.g., Reza et al.^21^.

**Table 1:** Surface area and pore properties of microalgae-derived hydrochars.

HTC temperature (°C)	Surface area (m^2^ g^−1^)	Pore volume (cm^3^ g^−1^)	Average pore width (nm)
180	15.3	0.10	27.1
220	32.1	0.16	20.3
260	51.2	0.34	26.4

### Physicochemical characterisation of hydrochars

The ash content of the hydrochars increased with increasing temperature (Table 2) as a result of the HTC treatment breaking down the organic biomass; this was released into the liquid phase, concentrating the inorganic content in the solid phase^37,38^.

**Table 2:** Yield and elemental composition (C, H, O, and N) of microalgae-derived hydrochars.

Sample	Yield (%)	C (%)	H (%)	O (%)	*N* (%)	Other elements (ash) (%)	Without ash				O: C	H: C
							C (%)	H (%)	O (%)	*N* (%)		
Initial	–	42.9	5.7	26.6	5.8	17.9	52.3	6.9	32.4	7.1	1.58	0.47
180 °C	46	36.8	4.5	21.1	3.3	34.3	56.0	6.9	32.1	5.0	1.46	0.43
220 °C	38	36.3	4.2	14.4	2.8	42.3	62.9	7.3	25.0	4.9	1.38	0.30
260 °C	26	36.7	4.2	9.3	2.9	46.9	69.1	7.9	17.5	5.5	1.36	0.19

The surface and bulk chemical compositions of the material before and after HTC treatment were investigated using X-ray photoelectron spectroscopy (XPS), diffuse reflectance infrared Fourier transform spectroscopy (DRIFTS), and elemental analysis (C, H, O, and N). Observation of elemental changes in the hydrochars during HTC provided insights into variations in chemical structure and carbonisation (Table 2). Since the ash content of the obtained materials obscured the changes in elemental composition, the proportions of carbon, hydrogen, nitrogen, and oxygen were normalised to the total content of these elements.

The proportion of carbon (Table 2) displayed a consistent increase with HTC processing temperature due to increasing aromaticity, as dehydration and decarboxylation reactions took place during the processing^39^. Nitrogen was reduced to approximately 5% of the original amount by HTC treatment at 180 °C due to hydrolysis of proteins and nitrogen compounds, including amino acids and ammonia^40^. A subsequent rise in the HTC temperature resulted in approximately a 7% reduction in the oxygen content for every increase of 40 °C, yielding a final oxygen content of 17.5% in the hydrochar produced at 260 °C. Here, reactive oxygen-containing groups were released from the feedstock, resulting in a carbon-rich hydrochar^41,42^. The hydrogen content remained relatively stable, decreasing slightly with increasing temperature, while the H: C atomic ratio decreased from 1.58 in raw algae biomass to 1.37 after HTC at 260 °C. The O: C ratio correspondingly changed, from 0.47 to 0.19, suggesting that HTC resulted in a more condensed aromatic (lower H: C ratio) and more carbonised (lower O: C ratio) structure^43^.

XPS showed that the surfaces of the microalgae biomass and hydrochar were primarily comprised of carbon, oxygen, and nitrogen, with trace quantities of several inorganic elements including calcium, phosphorous, aluminium, silicon, magnesium, and iron (Table 3). The speciation of these inorganic elements was ascertained from their peak positions, revealing their presence in forms such as CaCO_3_, Ca_3_(PO_4_)_2_, SiO_2_, Al(OH)_3_, Mg(OH)_2_, Fe(OH)_2_, and Fe(OH)_3_.

**Table 3:** Elemental composition of the surface, established using XPS.

Sample	C (at%)	O (at%)	*N* (at%)	Ca (at%)	*P* (at%)	Si (at%)	Al (at%)	Mg (at%)	Fe (at%)
Initial	68.2	24.8	4.5	1.0	0.6	0.6	0.3	–	–
180 °C	57.7	28.9	4.7	1.3	1.1	2.8	1.3	0.9	1.3
220 °C	59.1	27.4	4.01	0.6	0.5	3.7	1.6	1.4	1.6
260 °C	54.3	29.3	3.5	0.8	0.8	5.1	2.0	2	2.2

The initial algae biomass had 5.6 at% of oxygen bound to inorganic groups, and 19.2 at% in carbon-oxygen bonds. This ratio changed with increasing HTC temperature, resulting in 25.5% inorganic oxygen content after processing at 260 °C. Interestingly, the hydrothermal treatment caused the removal of calcium and phosphorous from the surface, along with the concentrating of other inorganic elements.

The removal of calcium and phosphorus correlates with the DRIFTS analysis of the elemental composition of the microalgae/hydrochars before and after HTC (Fig. 3), wherein we observed a band at 3620 cm^− 1^ with OH functionality, represented by the free OH groups^44^ of the inorganic components of the hydrochar^45^. Other bands representing inorganic components, e.g. bands associated with P-O stretching vibration of apatitic PO_4_^3−^ (606 and 560 cm^− 1^)^46^, increased with increasing HTC temperature. The characteristic band at 459 cm^− 1^ corresponded to the Mg–O stretching vibration^47^ resulting from the chlorophyll of microalgae.

**Fig. 2:**
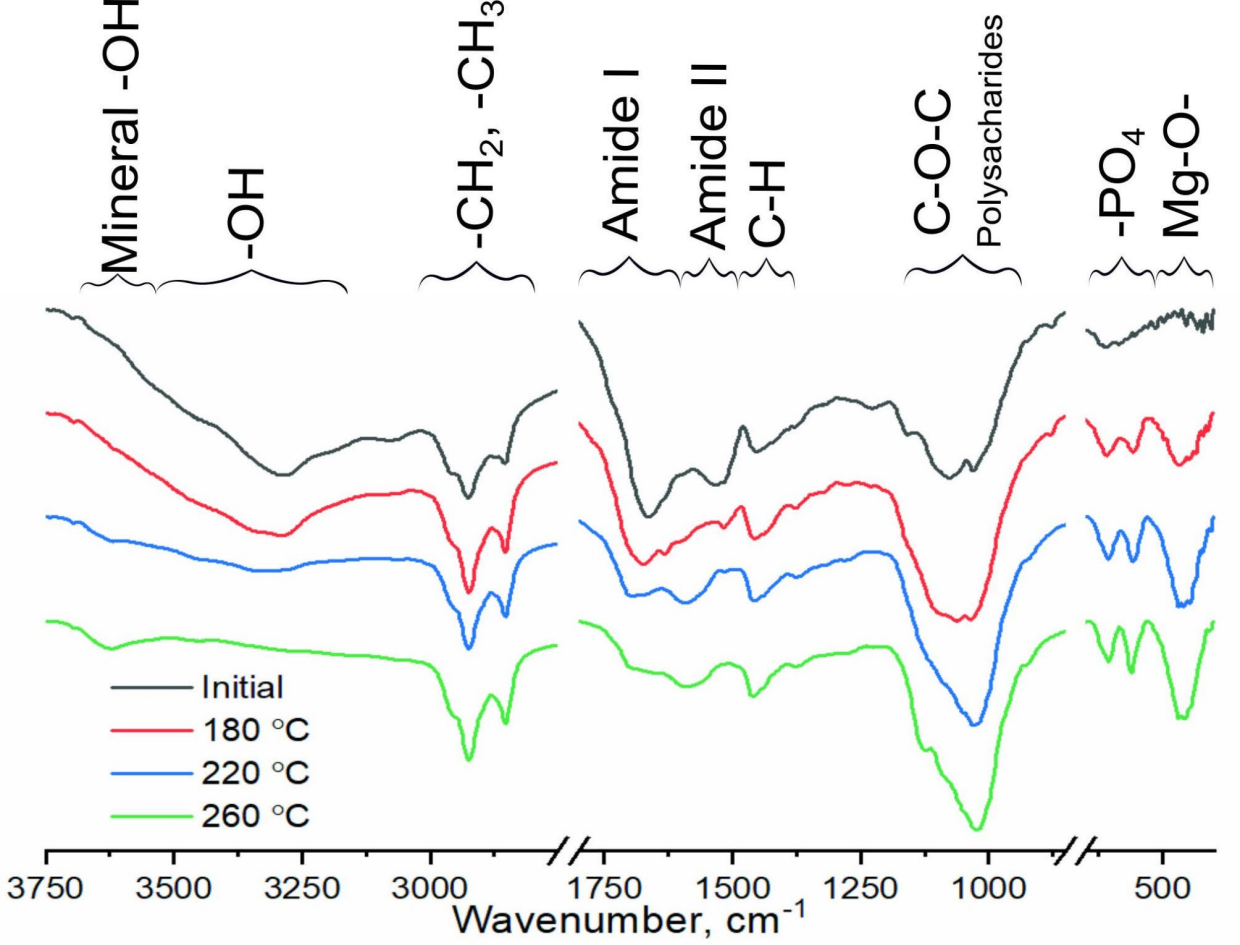
DRIFTS spectra of hydrochars prepared from microalgae.

Both XPS and DRIFTS were employed to examine the transformation of the organic components in the microalgae biomass as a result of the HTC process. The surfaces of both the raw microalgae biomass and the hydrochars were found to be constituted of a combination of alkane/alkene and aromatic structures, interspersed with various oxygen-containing functional groups, such as hydroxylic, carboxylic, and lactone. With increased temperature, there was an evident rise in the C-C/C = C/C-H bonds in the hydrochars (Table 4), which aligns with the common observation for organic biomass undergoing HTC treatment^42^. The increase in C-C/C = C/C-H also correlates with the DRIFTS analysis (Fig. 2), which showed the formation of aromatic C = C bonds, indicated by an increase in the 1600 cm^− 1^ band^48^ at higher HTC temperatures.

**Table 4:** Microalgae biomass and hydrochar carbon, oxygen, and nitrogen functionality as established using XPS spectra (at%).

Sample	Carbon				Oxygen			Nitrogen		
	C = C/C-C, C-H_x_	C-O-	C = O	COO^-^	O-C, Al(OH)_3_	O = C, CaCO_3_	Ar-OH	Fe-*N*	Organic *N*	Protonated *N*
	285.0 eV	286.6 eV	288.3 eV	289.5 eV	531.6 eV	532.6 eV	533.9 eV	398.6 eV	400.2 eV	401.9 eV
Initial	38.75	20.46	7.79	1.16	7.75	17.06	–	–	4.47	–
180 °C	36.96	14.30	5.31	1.12	19.50	9.41	–	0.42	3.86	0.44
220 °C	42.22	13.05	2.90	0.93	19.66	7.74	–	0.32	3.34	0.35
260 °C	45.98	6.14	1.58	0.58	22.82	5.47	0.98	0.66	2.49	0.37

XPS data further corroborate the loss of C-O-, C = O, and COO- groups from the surface of the microalgae biomass during HTC – a trend supported by the progressive decrease of bands in the 3000–3700 cm^− 1^ range due to dehydration. Concurrently, the carbohydrate-associated C-O-C band in the 1200–900 cm^− 1^ range remained nearly constant. Additionally, the XPS analysis indicated a decrease in organic nitrogen (400.2 eV), which aligns with the reduced intensity of protein amide bands (1670 cm^− 1^ and 1545 cm^− 1^) seen in Fig. 2.

HTC treatment of microalgae biomass can produce hydrochars with a range of properties that are potentially useful for the adsorption of organic compounds. The increase in surface area and pore volume with increasing HTC temperature suggests that the hydrochars became more porous and had larger surface areas available for adsorption. The increase in aromaticity and carbon content with increasing HTC temperature also suggests that the hydrochars became more hydrophobic, making them better able to adsorb non-polar organic compounds. The presence of inorganic elements could cause electrostatic interactions or chelation with organic compounds, further enhancing adsorption.

### Adsorption of contaminants of emerging concern using microalgae-derived hydrochars

The adsorption attributes of microalgae-derived hydrochars were explored by conducting water-based adsorption experiments on selected CECs with different physicochemical properties (Table S1 in the Supplementary Information). The principal criterion for the selection of CECs was a diverse range of hydrophilic and hydrophobic properties, which is believed to be paramount for adsorption onto porous carbon materials. To evaluate these properties, it is recommended that the distribution coefficient (*K*_*D*_) be utilised, as this factors in the specific ionic form of a compound at a given pH^49,50^.

**Fig. 3:**
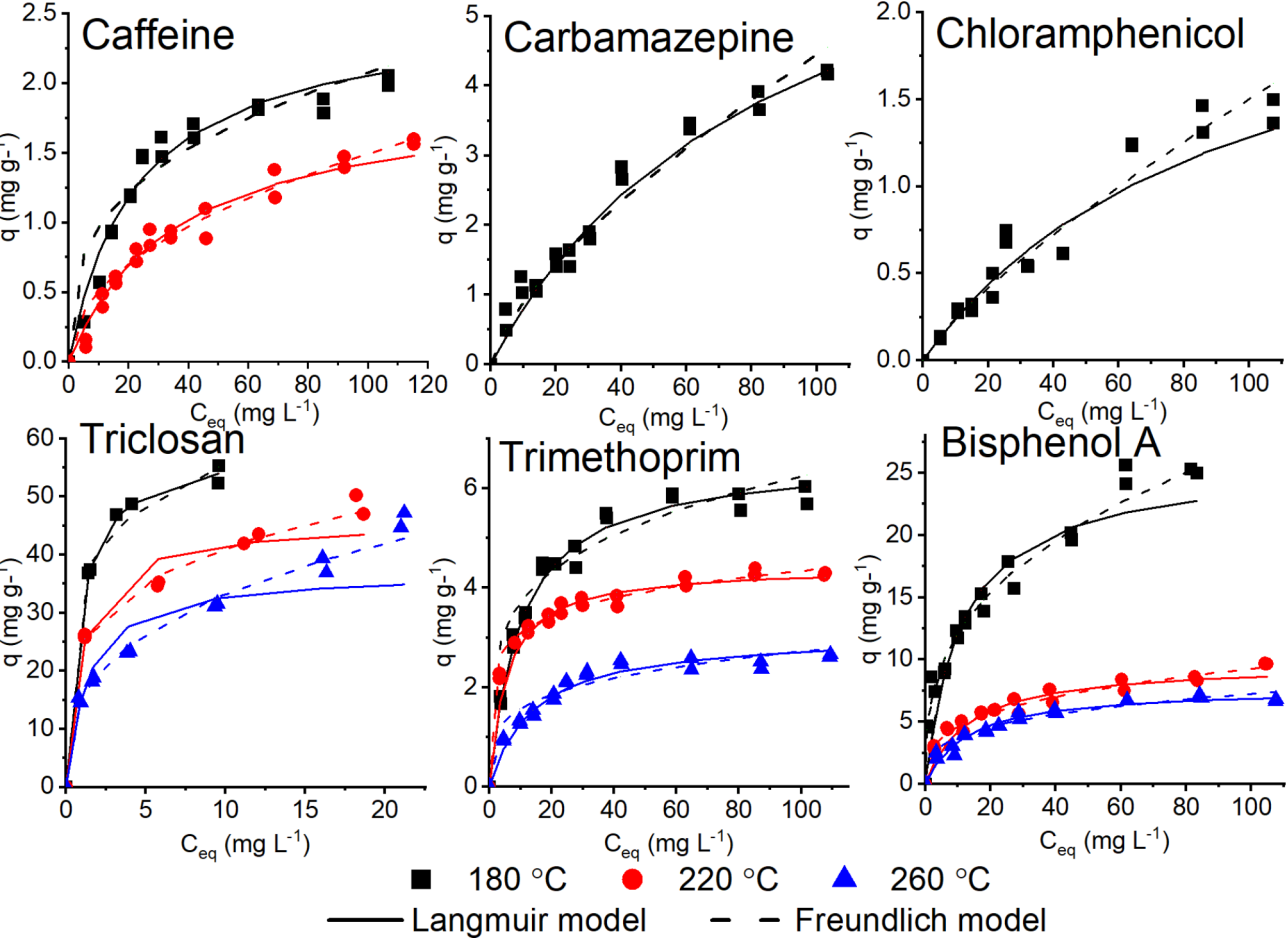
Isotherms showing the adsorption of caffeine, trimethoprim, chloramphenicol, carbamazepine, bisphenol A, and triclosan on microalgae-derived hydrochars.

The batch adsorption experiments yielded adsorption isotherms (Fig. 3) that exhibited consistent characteristics. Specifically, they showed a substantial increase in adsorbed quantities at lower CEC concentrations, a decrease around equilibrium concentration, and a progressively slower decrease at higher concentrations. These adsorption isotherms align with the L-type according to the Giles classification^51^. L-type isotherms denote the monomolecular-layer adsorption of CEC molecules on the surfaces of sorbents, suggesting minimal competition between CEC molecules and water molecules for active adsorption sites on the surface^52^.

The correlation coefficients (R^2^ values) for both the Langmuir and Freundlich adsorption models predominantly exhibited high values (> 0.75). This high correlation indicates an excellent fit of the models to the experimental data. However, a closer examination of the R^2^ values (Table 5) reveals that the Langmuir model provides a superior fit to the experimental equilibrium data in most cases as compared to the Freundlich model. This observation suggests that the adsorption of the studied CECs predominantly occurs at specific homogeneous sites on the adsorbent surface: once a molecule occupies such a site, it prohibits any further adsorption, leading to the formation of a monomolecular layer^53^. In this study, our analysis was primarily centred on cases where the maximum adsorption (*q*_*max*_), as predicted by the Langmuir model, was greater than 1 mg g^− 1^. Cases demonstrating lower adsorption were not included in Fig. 3, and were marked as ‘na’ (‘no adsorption’) in Table 5.

**Table 5:** Adsorption properties of hydrochars produced from microalgae relative to the investigated CECs.

Substance	HTC temperature (°C)	Models of adsorption						*ΔG*^*0*^ (kJ mol^−1^)
		Langmuir			Freundlich			
		*q*_*max*_ (mg g^−1^)	*K*_*L*_, (L g^−1^)	R^2^	*K*_*F*_ (L mg^−1^)	n	R^2^	
Caffeine	180	2.5	0.04	0.94	0.5	3.0	0.86	− 15.6
	220	1.9	0.03	0.94	0.2	2.1	0.94	− 14.7
	260	na	na	na	na	na	na	na
Trimethoprim	180	6.6	0.10	0.94	2.2	4.4	0.94	− 21.4
	220	4.4	0.19	0.90	2.2	6.9	0.75	− 21.9
	260	3.1	0.07	0.90	0.9	4.29	0.75	− 15.8
Chloramphenicol	180	2.5	0.01	0.94	0.04	1.3	0.95	− 13.6
	220	na	na	na	na	na	na	na
	260	na	na	na	na	na	na	na
Carbamazepine	180	7.8	0.01	0.96	0.18	1.4	0.96	− 16.5
	220	na	na	na	na	na	na	na
	260	na	na	na	na	na	na	na
Bisphenol A	180	25.9	0.09	0.96	5.4	2.9	0.97	− 24.4
	220	9.7	0.08	0.87	2.3	3.3	0.91	− 20.6
	260	7.7	0.08	0.91	2.0	3.6	0.83	− 18.3
Diclofenac	180	na	na	na	na	na	na	na
	220	na	na	na	na	na	na	na
	260	na	na	na	na	na	na	na
Triclosan	180	58.8	1.18	0.99	35.4	5.1	0.94	− 32.7
	220	45.6	1.08	0.91	24.5	4.4	0.98	− 30.8
	260	37.1	0.74	0.88	15.4	3.0	0.98	− 27.5

‘na’ is no adsorption (adsorption is less than 1 mg g^− 1^).

The adsorption capacity of the hydrochars exhibited considerable variation based on the HTC temperature and type of the CECs. Triclosan exhibited the highest adsorption capacity (*q*_*max*_) at 180 °C, 58.8 mg g ^− 1^, which gradually reduced to 37.1 mg g^− 1^ at 260 °C. Bisphenol A and trimethoprim also demonstrated substantial adsorption at 180 °C; like triclosan, however, adsorption decreased with increasing HTC temperature. Caffeine, chloramphenicol, and carbamazepine showed no adsorption at 260 °C, and diclofenac did not display any adsorption for any of the tested hydrochars. The Langmuir constant *K*_*L*_ provides valuable insights into the affinity between the adsorbent surface and the adsorbate molecules. In our study, triclosan exhibited the highest *K*_*L*_ values (1.18, 1.08, and 0.74 L g^− 1^ for hydrochars produced at 180, 220, and 260 °C respectively), indicating strong binding affinity between triclosan and the hydrochar surface. The observed decrease in *K*_*L*_ values with increasing HTC temperature suggests that higher carbonization temperatures reduce the binding affinity, likely due to the reduction in oxygen-containing functional groups. This trend aligns with the observed *q*_*max*_ values and supports our finding that surface functionality plays a crucial role in adsorption performance. For compounds with lower *K*_*D*_ values, such as caffeine and chloramphenicol, the significantly lower *K*_*L*_ values (0.04 and 0.01 L g^− 1^ respectively at 180 °C) indicate weaker binding affinity, explaining their reduced adsorption capacity.

The Freundlich model constants (*K*_*F*_), which offer insights into adsorption capacity and intensity, revealed an interesting pattern in this study. Triclosan stands out for having the highest *K*_*F*_ value (35.4 L mg^− 1^) at 180 °C, indicating a strong adsorption process. However, much like the patterns described above, the *K*_*F*_ values displayed a tendency to diminish as the HTC temperature increased for most of the studied CECs, suggesting that increased HTC processing temperature reduces the adsorption capacity of the hydrochars for these substances. It also means that the increase in surface area with higher HTC temperature (Table 2) did not increase the adsorption of the investigated CECs. Along with the adsorption capacity, the Freundlich constant, *n*, plays a significant role in describing the adsorption process: it is an indicator of the favourability of adsorption, with values over 1 and less than 10 denoting a favourable process, a value of 1 signifying irreversible adsorption, and anything less than 1 indicating unfavourable adsorption^54^. This study resulted in a set of *n* values that clearly depict the uptake of CECs as a favourable adsorption process for each of the tested substances, except for diclofenac. All the values were within the favourable range across different temperatures, signifying that the affinity of the hydrochars for these substances is quite strong, and the adsorption process is not easily reversed.

The change in Gibbs free adsorption energy, denoted as *-ΔG*^*0*^, consistently had negative values. This negative shift in Gibbs free energy suggests the occurrence of spontaneous physisorption processes^55^. When physisorption occurs, the change in standard free energy varies in the range of -20 to 0 kJ mol^− 1^, while for chemisorption the standard free energy varies in the range of -80 to -400 kJ mol^− 1^^56^. The *-ΔG*^*0*^ values for the CECs tested in this study (-13.6 to -32.7 kJ mol^− 1^) suggest that the adsorption process was heterogeneous, but that primarily physisorption took place. Hydrochars are composed of a variety of different functional groups, including aliphatic, aromatic, and oxygen-containing groups. These functional groups can interact with the adsorbate molecules in different ways, resulting in a mixture of physisorption and chemisorption.

In this study we investigated the simultaneous adsorption of six organic compounds (caffeine, trimethoprim, chloramphenicol, carbamazepine, bisphenol A, and diclofenac). This approach was taken on the basis that: it is more realistic than studying the adsorption of a single compound, as real-world systems often contain multiple contaminants; the adsorption behaviour of the contaminants may have been different due to multiple being present; and they were competing for the same adsorption sites on the adsorbent. For example, another study found the adsorption of bisphenol A using an argan nutshell hydrochar from a single-component solution to be 1162.7 mg g^− 1^^57^ (25.9 mg g^− 1^ in this study). Concurrent adsorption of organic compounds from a solution refers to the simultaneous attachment of various organic compounds to the adsorbent. This process is complex, as it involves competition between multiple organic compounds for the same adsorption sites on the adsorbent^58,59^.

When evaluating adsorption, substances with higher *K*_*D*_ values could be expected to have higher adsorption on carbon materials due to their greater affinity for nonpolar environments. Han et al.^60^ state that the amorphous carbon-alkyl domains of hydrochars are the primary reason for their high sorption capacity with regard to hydrophobic compounds. It was observed in the present study that adsorption of the relatively hydrophobic bisphenol A and triclosan decreased for hydrochars with lower carbon content. Based on this premise, triclosan – with the highest *K*_*D*_, of 5.13 – had the highest *q*_*max*_ and *K*_*F*_ values of all of the hydrochars, demonstrating superior adsorption potential. Bisphenol A, with the second-highest *K*_*D*_, of 3.63, also had high *q*_*max*_ and *K*_*F*_ values, again validating the idea of *K*_*D*_-based prediction. Interestingly, carbamazepine had a *K*_*D*_ of 2.28 and did not exhibit any adsorption at 220 °C and 260 °C, suggesting that factors other than *K*_*D*_ (such as chemical structure and hydrochar characteristics at these temperatures) influence adsorption.

The adsorption of compounds with lower *K*_*D*_ values was higher for hydrochars produced at 180 °C (caffeine 2.5 mg g^− 1^, trimethoprim 6.6 mg g^− 1^, chloramphenicol 2.5 mg g^− 1^, and carbamazepine 7.5 mg g^− 1^) than for the hydrochars produced at higher temperatures; for the hydrochars produced at 260 °C, adsorption occurred only for trimethoprim (3.1 mg g^− 1^). The hydrochars produced at lower temperatures (180 and 220 °C) contained more negatively charged oxygen-containing functional groups (Fig. 2), and more efficiently adsorbed positively charged cation molecules such as trimethoprim. Such a mechanism of adsorption of cationic molecules (in the form of blue methylene dye) has been observed elsewhere for microalgae-derived hydrochars^61^. The negatively charged molecules in chloramphenicol had low (2.5 mg g^− 1^ for chloramphenicol and hydrochar prepared at 180 °C) adsorption, and those in diclofenac had no adsorption, due to repulsion of the negatively charged functional groups on the surface of the hydrochars.

While the *K*_*D*_ values seem to suggest a general trend for the adsorption potential of these organic compounds, there were exceptions. These exceptions indicate that adsorption may be influenced by other factors, including the HTC processing conditions, the characteristics of the hydrochar, and the chemical structure of the compound to be adsorbed. More detailed studies are needed in order to fully understand the influence of these factors on adsorption.

## Conclusions

This study demonstrates the novel application of microalgae-derived hydrochars for removing multiple CECs simultaneously from aqueous systems. Unlike previous single-compound studies, our multi-component approach provides insights into real-world adsorption scenarios. We established that surface functionality, rather than surface area, primarily determines adsorption performance - challenging conventional assumptions about adsorbent design.

The surfaces of both raw microalgae biomass and the produced hydrochars were characterized by alkane/alkene and aromatic structures with diverse oxygen-containing functional groups. Our findings reveal that HTC temperature significantly influences these surface characteristics, with higher temperatures increasing alkane/alkene and aromatic structures while reducing oxygen- and nitrogen-containing groups. This understanding enables targeted hydrochar production for specific contaminant removal applications.

Compounds with higher *K*_*D*_ values, such as bisphenol A and triclosan, adsorbed more readily to hydrochars in comparison to more hydrophilic CECs. Adsorption proved to be most effective with hydrochars prepared at 180 °C, which also had better capacity for the adsorption of more hydrophobic compounds. Additionally, the hydrochars that exhibited a lesser degree of carbonisation more efficiently adsorbed positively charged cationic molecules, such as trimethoprim, due to the higher concentration of negatively charged oxygen-containing functional groups. Surface area proved to have very little influence on adsorption.

The results of this study provide valuable insights regarding the adsorption of CECs using microalgae-derived hydrochars. There are still questions that need to be addressed in future studies, such as the effect of the pH or ionic strength of the solution on the adsorption of CECs.

## Materials and methods

###  Raw algae biomass

A microalgae polyculture (*Scenedesmus*, *Desmodesmus*,* Chlorella*, *Cosmarium*, and cocci and bacilli bacteria) was provided by the Swedish University of Agricultural Science (Umeå, Sweden). The microalgae were grown in an open pond fed with municipal wastewater (Vakin, Umeå, Sweden), while heat and power-plant flue gases (Umeå Energi AB, Umeå) were used for pH regulation and carbon. Temperature and light were not controlled, and natural variation took place over the growth season, between April and November. Microalgae were harvested weekly by sedimentation; this was centrifuged at 5000 rpm (US Filtermaxx, Jacksonville, Florida, USA) to 15 wt% solids, then stored in a freezer at -20 °C until use^62^.

### Hydrothermal carbonisation

In a previous study^61^, temperature was found to be the parameter that most strongly affected the properties of hydrochar made using microalgae (in terms of specific surface area, pore volume, ash content, surface functionality, and methylene blue adsorption capacity); on that basis, it was the process parameter that was explored in this study.

The HTC treatment was performed with six replicates per temperature, according to the following protocol: 10 g of homogenised microalgae biomass (15 wt% solids) was loaded into a 25 mL polytetrafluoroethylene-lined stainless-steel autoclave reactor with 8.0 mL of deionised water. The reactor was heated to 180, 220, or 260 °C at an average heating rate of 6 °C/min and this nominal temperature was held for six hours, after which the reactor was allowed to cool to room temperature. Solid and liquid products were separated by filtering using a 0.45 μm PTFE filter (Whatman, Thermo Fisher Scientific), and the filter cake was washed with 100 mL of deionised water, 20 mL of acetone (analytical grade), and another 100 mL of deionised water. Lastly, the solid product was oven-dried at 105 °C.

Hydrochar yield (Y) was calculated using Eq. 1:

1 $$\:{Y}_{1}\:\left(\text{\%}\:db\right)=\:\frac{{m}_{hydrochar}}{{m}_{feed\:}}\:\times\:100$$

where *m* is the mass (g) of the dry sample.

### Structural determination and morphology

Final analysis of the raw algae biomass and hydrochar samples was undertaken using a CHNS-O EA3000 elemental analyser (Eurovector Srl, Italy). The ash content was calculated by subtracting the CHNS-O content from the total mass. SEM analysis was performed on a ZEISS EVO scanning electron microscope operated in low-vacuum mode (50 Pa) to assess the morphology of the product.

### XPS analysis

XPS spectra were acquired using a Kratos Axis Ultra DLD electron spectrometer with a monochromated Al Kα source operated at 150 W, a hybrid lens system with a magnetic lens providing an analysis area of 0.3 × 0.7 mm, and a charge-neutralisation system. The binding energy (BE) scale was referenced to the C1s line of sp^2^ hybridisation, set at 284.6 eV. The processing of the spectra was accomplished using the Kratos and CasaXPS software packages. The curve fitting of the high-resolution C1s, O1s, N1s, and Fe2p spectra was performed after Shirley background subtraction with a minimum number of spectral components. The BE position and full width at half maximum (FWHM) of the components were not fixed.

### DRIFTS analysis

DRIFTS spectra were acquired using a Bruker IFS 66 v/S FT-IR spectrometer (vacuum bench) coupled with DTGS and MCT detectors. Hydrochars were mixed with KBr in a ratio of 1:12 and placed in sample holders. The measurements were performed under vacuum conditions (below 0.7 kPa), covering the spectral range 4000–400 cm^− 1^ at 2 cm^− 1^ resolution. Spectra were baseline corrected using a linear technique, with automatic point selection and min–max normalisation over the entire spectral range using the KnowItAll software (John Wiley & Sons).

### Surface area and porosity measurements

Surface area and pore volume were determined using the Brunauer–Emmett–Teller (BET)^63^ and Barrett–Joyner–Halenda (BJH) methods^64^ on a TriStar 3000 automated nitrogen sorption/desorption instrument (Micromeritics, Norcross, GA, USA). In brief, 0.1 g of dried hydrochar was degassed under N2 flow at 120 °C for 2 h. Micropore surface area, external surface area, and micropore volume were calculated using the t-plot method^65^.

### Adsorption

Batch adsorption experiments were carried out to investigation the adsorption of six CECs that can be found in surface water influenced by human activity, and that may have adverse health effects, using the hydrochars^66–71^. The selected CECs span a wide range of distribution coefficient (*K*_*D*_), where *K*_*D*_ is the pH-dependant differential solubility in octanol/water^49^, and is thought to relate to the sorption of organic compounds on carbon materials^49^. The CECs were: chloramphenicol (antibiotic; *K*_*D*_ = 1.02), trimethoprim (antibiotic; *K*_*D*_ = 1.00), carbamazepine (anticonvulsant; *K*_*D*_ = 2.28), diclofenac (anti-inflammatory drug; *K*_*D*_ = 1.17), bisphenol A (industrial chemical; *K*_*D*_ = 3.63), triclosan (personal care product; *K*_*D*_ = 5.13), and caffeine (stimulant; *K*_*D*_ = 0.28). Further information on the CECs can be found in Table S1 in the Supplementary Information.

A mixed CEC stock solution with the concentration 5 g·L^− 1^ (3 g·L^− 1^ for triclosan due to low water solubility) was prepared in methanol and diluted in ultrapure water (MilliQ) to 5.0; 7.0; 10.0; 12.0; 15.0; 20.0; 30.0; 40.0; 80.0; and 100.0 mg·L^− 1^ (3.0; 4.2; 6.0; 7.2; 9.0; 12.0; 18.0; 24.0; 48.0; and 60.0 mg L^− 1^ for triclosan). The adsorption experiments were performed in duplicates for each concentration, and conducted at 20 °C in HDPE tubes containing 50 mg of hydrochar and 50 mL of mixed CEC solution. pH was adjusted to 7.0 using 1 mol·L^− 1^ of NaOH solution. The tubes were shaken using an orbital shaker at 50 rpm for 24 h, after which the CEC solution was filtered using 0.45 μm nitrocellulose membrane syringe filters (Filtropur, Sarstedt).

Concentrations of CECs in solution were determined using UV-absorption measurements on a HP 1100 Chromatography System (Agilent, Germany) with a Hypersil ODS 5 μm C18, 100 × 2.1 mm columns. The column temperature was kept at 30 °C, and the mobile phase was acetonitrile and 0.1 mol·L^− 1^ ammonium acetate buffer (VWR, Belgium) at a flow rate of 0.5 ml·min^− 1^. The injection volume was 10 µL, and detection was performed at 254 nm for caffeine, trimethoprim, chloramphenicol, carbamazepine, and diclofenac, and at 280 nm for triclosan. Bisphenol A was detected with a fluorescence detector at an excitation wavelength of 280 nm, and emission at 340 nm.

The equilibrium adsorption (*q*_*eq*_) was calculated as follows:

2 $$\:{q}_{eq}=\frac{\left({C}_{0}-{C}_{eq}\right)\cdot\:V}{m}$$

where *С*_*0*_ (initial) and *С*_*eq*_ (equilibrium) are the concentrations of the CEC (mg·L^− 1^); *V* is the solution volume (L); and *m* is the mass of sorbent (g).

The maximal adsorption (*q*_*max*_) was calculated from the linear form of the Langmuir model of adsorption^72^:

3 $$\:\frac{1}{{q}_{eq}}=\frac{1}{{q}_{max}}+\frac{1}{{q}_{max}{K}_{L}{C}_{eq}}$$

where *q*_*max*_ is maximum adsorption (mg·g^− 1^) and *K*_*L*_ is the Langmuir isotherm constant (L·mg^− 1^).

The values for *q*_*max*_ and *K*_*L*_ were calculated using the intercept and slope of the Langmuir plot of *1/C*_*eq*_ versus *1/q*_*eq*_.

Assessment of the adsorption characteristics of the hydrochars was performed using the Freundlich model of adsorption^73^:

4 $$\:{q}_{eq}={K}_{F}{C}_{eq}^{1/n}$$

where *K*_*F*_ and *n* are the Freundlich constants for a given adsorbate and adsorbent at a particular temperature.

The “conventional component” method used by e.g., Kozyatnyk, et al.^74^ was used for the determination of the change in Gibbs free energy of adsorption. The equation for the isotherm of adsorption of an organic compound from an aqueous solution can be represented as:

5 $$\:\frac{{V}_{a}}{{a}_{i}{\phi\:}_{i}}=\left({V}_{i}-{V}_{{H}_{2}O}\right)-\frac{1}{{K}_{i}{C}_{i}}$$

where *a*_i_ is the specific value of the *i*-th component’s equilibrium adsorption at the concentration *C*_i_, expressed in terms of the molar concentration; *V*_a_ is the volume of the adsorption space of the sorbent; *K*_i_ is a constant for a selective-adsorption isotherm; *φ*_i_ is the activity coefficient of the *i*-th component; and *V*_i_ and *V*_*H*2*O*_ are the molar volumes of the adsorbed components. For a low-concentration solution, *φ*_i_ is only slightly different from unity. Therefore, the graphical representation of *V*_a_*/a*_i_ versus *1/C*_i_ allows *K*_i_ to be determined as the cotangent of the angle of the straight line to the ordinate axis. From this, the change in Gibbs free adsorption energy is determined using Eq. 6:

*-ΔG*^*0*^_*a*_ *= R T lnK*_*i*_ (6).

where *-ΔG*^*0*^_*a*_ is the change in Gibbs free adsorption energy; *R* is the universal gas constant; and *T* is the absolute temperature.

## Electronic supplementary material

Below is the link to the electronic supplementary material.

Supplementary Material 1

## Data Availability

All relevant data generated or analysed during this study are included in this published article and in the supplementary information. Additional parts of the datasets used and/or analysed during the current study are available from the corresponding author upon request (CC-BY).
