# Supplementary material for: Adsorption of organic contaminants of emerging concern using microalgae-derived hydrochars
Source: Sci Rep. 2025 Mar 17;15:9059. doi: 10.1038/s41598-025-92717-y (PMC11914538; doi:10.1038/s41598-025-92717-y)
Supplement: Supplementary file 1 — Supplementary Material 1 [file 41598_2025_92717_MOESM1_ESM.pdf]

Supplementary information

Adsorption of organic contaminants of emerging concern using microalgae-derived hydrochars

Ivan Kozyatnyk[1,2], Veronica Benavente[1,3], Eva Weidemann[1], Stina Jansson[1, *]

[1] Department of Chemistry, Umeå University, Linnaeus väg 6, SE-901 87 Umeå, Sweden
[2] Department of Health, Medicine and Caring sciences, Unit of Clinical Medicine, Occupational and Environmental Medicine,
Linköping University, 581 83 Linköping, Sweden
[3] RISE Processum AB, Hörneborgsvägen 10, SE-891 22 Örnsköldsvik, Sweden

*Corresponding Author

The Supplementary Information contains one figure, Figure S1, accompanied with an in-depth discussion of the nitrogen adsorption-desorption isotherm, and one table, Table S1, which details physiochemical properties of the CEC used in this study.

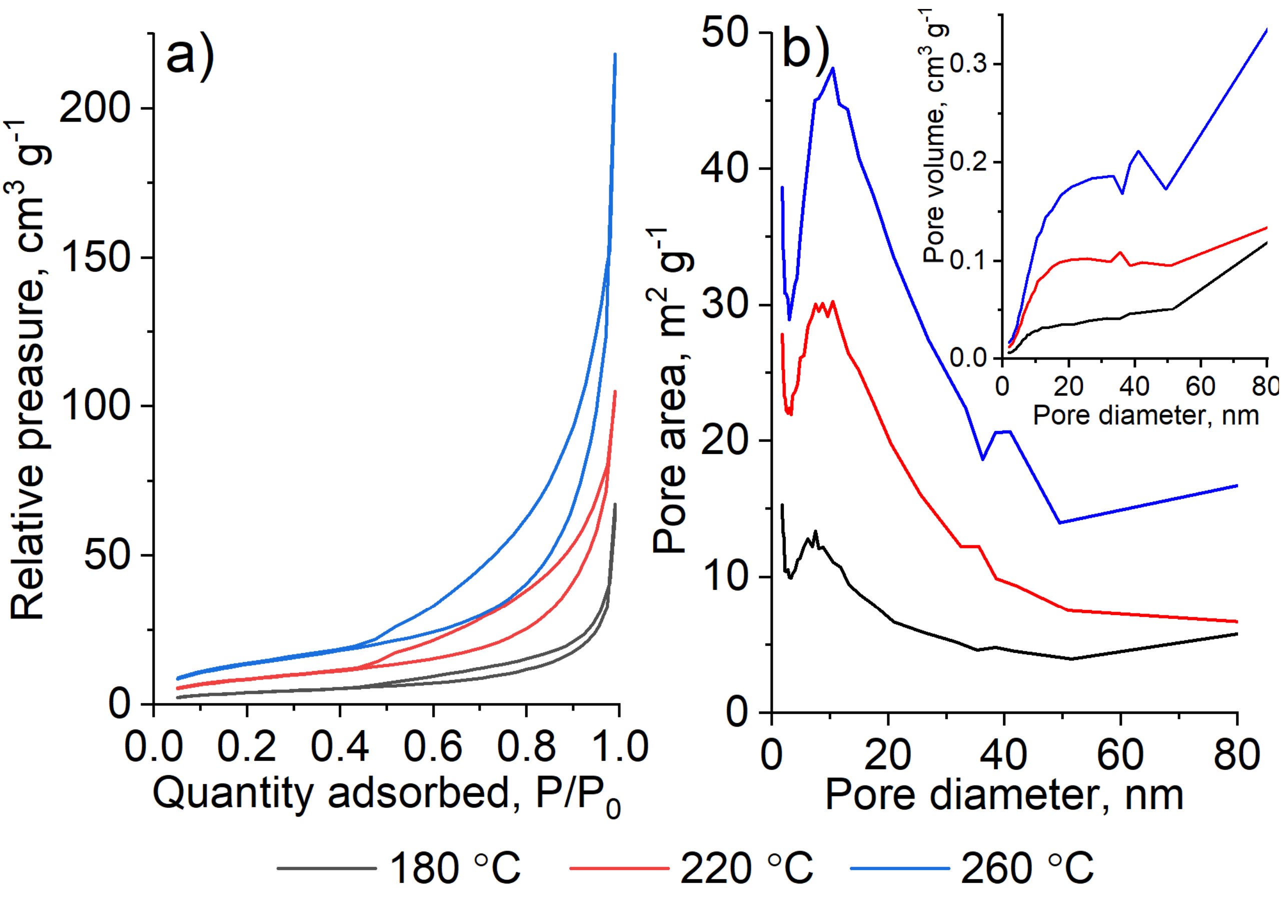

Fig. S1. Nitrogen adsorption-desorption isotherm (a) and pore area and volume distribution (b) of microalgae hydrochars

The nitrogen adsorption-desorption isotherm of the resulting microalgae hydrochars exhibits type II isotherms, reflecting a reversible adsorption-desorption process (see Fig. S1). This behaviour, characterized by vigorous adsorption at relative pressures near unity, is typical for gases undergoing physisorption on nonporous or macroporous adsorbents, and can be ascribed to the capillary condensation of nitrogen in the substantial voids of the macropores. Hysteresis loops were observed in the relative pressure range of 0.45 to 0.99 for all hydrochars, demonstrating type H3 characteristics. This type of hysteresis loop is frequently associated with non-rigid conglomerates of plate-like particles. However, it can also occur when the pore network is composed of macropores or large mesopores that lack pore condensate [1]. A noticeable increase in the hysteresis loop area for hydrochars created at higher temperatures suggests a growing presence of mesopores, as evidenced by the pore size distribution curve of the algae hydrochars (Fig. S1). A broad pore size distribution was observed within the mesopore region, ranging from 10 to 50 nm, and peaking at 10 nm. Interestingly, the proportion of pores with a 10 nm diameter increase covaries with increasing HTC processing temperature.

Table S1. Properties of studied organic compounds

Property	Database	Caffeine	Chloram-phenicol	Trimetho-prim	Carba-mazepine	Bisphenol A	Diclofenac	Triclosan
Structure	ChemSpider	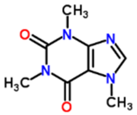	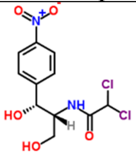	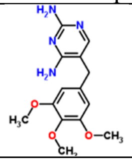	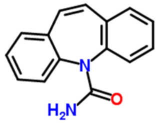	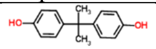	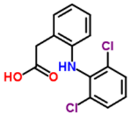	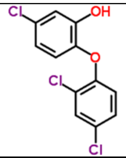
Molecular Weight, g/mol	PubChem	194.194	323.126	290.323	236.274	228.291	296.147	289.536
Molar Volume, cm3	ChemSpider (ACD/Labs)	133.4	208.8	231.9	186.6	199.6	206.8	194.3
Hydrogen Bond Donor Count	PubChem	0	5	2	1	2	2	1
Hydrogen Bond Acceptor Count	PubChem	3	5	7	1	2	3	2
Rotatable Bond Count	ChemSpider (ChemAxon)	0	6	5	0	2	4	2
Number of Rings	ChemSpider (ChemAxon)	2	1	2	3	2	2	2
Topological Polar Surface Area, A^2	PubChem	58.4	115	106	46.3	40.5	49.3	29.5
Boiling Point, C	PubChem	178	644.9	526	411	360.5	412	120
Melting Point	PubChem	237	150.5	202	190.2	153	284	57
Density, g/cm³	PubChem	1.23	1.5	1.3	1.3	1.2	1.4	1.5
Vapor Pressure, mm Hg	PubChem	0.0000009	1.73E-12	0	0.000000184	0.00000004	6.14E-08	0.0000046
Water Solubility, mg/L	PubChem	21600	2500	400	17.7	300	2.37	10
LogS	PubChem	-0.97	-2.11	-2.86	-3.2	-3.4	-4.8	-4.7
pKa (Strongest Acidic)	ChemSpider (ChemAxon)		7.49	17.33	15.96	9.78	4	7.68
pKa (Strongest Basic)	ChemSpider (ChemAxon)	-0.92	-2.8	7.16	-3.8	-5.5	-2.1	-6.7
Dissociation Constant pKa	PubChem	14	9.75	7.12	13.9	9.6	4.15	7.9
Kovats Retention Index	PubChem	1760	2310	2645	2296	2108	2495	2114
Henrys Law Constant, atm-m3/mole	ChemSpider (EPISuite)	7.116E-13	1.915E-15	2.39E-014	1.549E-09	3.948E-10	5.296E-09	3.834E-07

Property	Database	Caffeine	Chloram-phenicol	Trimetho-prim	Carba-mazepine	Bisphenol A	Diclofenac	Triclosan
Log Octanol-Water	PubChem	-0.07	1.02	0.91	2.673	3.431	4.058	4.76
Log Octanol-Air	ChemSpider (EPISuite)	8.765	17.169	12.920	10.805	12.747	14.224	11.45
Soil Adsorption Coefficient Log Koc	ChemSpider (EPISuite)	1	1	2.957	3.588	4.876	2.921	4.265
Distribution coefficient Log D (pH 7.4)	ChemSpider (ACD/Labs)	0.28	1.02	1.00	2.28	3.63	1.17	5.13
Polarizability, cm3	ChemSpider (ACD/Labs)	2E-23	2.88E-23	3.18E-23	2.76E-23	2.7E-23	3.03E-23	2.75E-23
Surface Tension, dyne/cm	ChemSpider (ACD/Labs)	55.8	66.1	53.9	57.3	46	58	51.7

References

[1] Thommes, M., Kaneko, K., Neimark, A.V., Olivier, J.P., Rodriguez-Reinoso, F., Rouquerol, J. and Sing, K.S.W. 2015. Physisorption of gases, with special reference to the evaluation of surface area and pore size distribution (IUPAC Technical Report). Pure Appl. Chem. 87(9-10), 1051-1069.
